# Immunohistochemical Expression of Tenascin-C in Canine Meningiomas

**DOI:** 10.3390/vetsci11100462

**Published:** 2024-10-01

**Authors:** Fabiano José Ferreira de Sant’Ana, Ester Blasco, Martí Pumarola

**Affiliations:** 1 Laboratório de Diagnóstico Patológico Veterinário (LDPV), Universidade de Brasília (UnB), Brasília-Distrito Federal 70636-200, Brazil; 2 Unitat de Patologia Murina i Comparada (UPMiC), Departament de Medicina i Cirurgia Animals, Facultat de Veterinària, Universitat Autònoma de Barcelona, Bellaterra, 08193 Barcelona, Spain; ester.blasco@uab.cat (E.B.); marti.pumarola@uab.cat (M.P.)

**Keywords:** neuropathology, meningioma, oncology, TN-C

## Abstract

**Simple Summary:**

Many studies have analyzed the relationship between the different subtypes of canine meningiomas and prognostic immunohistochemical markers. However, the expression of tenascin-C (TN-C), an extracellular matrix glycoprotein, in canine meningiomas has not been performed. Twenty-one cases of canine meningioma were analyzed. The immunoreactivity of TN-C was mild in grade I, moderate in grade II, and moderate to severe in grade III neoplasms. Immune positivity was observed in the stroma and perivascular space in all subtypes. In addition, the concentric whorls of neoplastic cells were labeled positive in some psammomatous and transitional meningiomas. The reaction to TN-C was more significant in grade II and III tumors. The immunohistochemical findings of the current study suggest that TN-C can act as a stromal marker, mainly in grade II or III meningiomas.

**Abstract:**

In humans, tenascin-C (TN-C) expression has been detected in more aggressive neoplasms of the central nervous system, such as gliomas and meningiomas. No study has analyzed the immune expression of TN-C in canine meningioma. The current study aimed to investigate the immunohistochemical distribution of TN-C in different grades of canine meningiomas. Twenty-one cases of canine meningioma (12 grade I, 6 grade II, and 3 grade III) were analyzed. All samples were examined by immunohistochemistry with the following antibodies: TN-C, epithelial membrane antigen (EMA), Ki-67, pan-cytokeratin (Pan CK), and vimentin. The histopathological diagnosis of meningioma was reinforced with the positive labeling of vimentin (moderate to strong) and EMA (mild to moderate) in neoplastic cells in most cases, independently of its grade or subtype. The immunoreactivity of TN-C was irregular: mild in grade I, moderate in grade II, and moderate to severe in grade III neoplasms. Usually, immune positivity was observed in the stroma and perivascular space in all subtypes. In addition, the concentric whorls of neoplastic cells were labeled positive in some psammomatous and transitional meningiomas. The reaction to TN-C was more significant in grade II and III tumors. The immunohistochemical findings of the current study suggest that TN-C can act as a stromal marker, mainly in grade II or III meningiomas.

## 1. Introduction

Meningioma is the most commonly diagnosed neoplasm affecting the central nervous system of dogs [1,2,3,4]. It arises from meningothelial cells of the arachnoid layer. This tumor presents a great diversity of histologic patterns due to a double contribution of mesodermal and ectodermal neural crest components to developing the leptomeninges [5]. Approximately 82% of meningiomas have an intracranial location, 15% intraspinal, and 2–3% retrobulbar [1,3,6]. The olfactory bulb and frontal cortex are regions commonly affected by this neoplasm, but it can occur anywhere of leptomeninges [1]. In addition, there are descriptions of paranasal meningiomas affecting dogs [7]. Clinical signs are variable according to anatomical localization of the neoplasm and the compressed region of the nervous system and include conscience changes, seizures, and vestibular dysfunctions [8,9]. Grossly, meningiomas consist of well-demarcated, usually lobulated, firm nodules or masses that compress or infiltrate adjacent nervous tissue. Histologically, canine meningioma shows morphological similarities with its human counterparts. Thus, meningiomas are classified into grade I, II (atypical), and III (malignant) [1,10].

Although this neoplasm is commonly diagnosed and studied in humans and animals, many pathogenic details and biological behavior still need better clarification. The knowledge of the responsible mechanisms for the progression of intracranial meningiomas is an important prerequisite for the development of effective therapeutic strategies, as well as for estimates of the survival and prognosis of affected animals. Numerous studies have been performed in recent decades to analyze the relationship between the different graduations of meningiomas and prognostic immunohistochemical markers [1]. For example, the proliferative activity by Ki-67 or PCNA has been evaluated in some studies [11,12,13], as well as the expression of hormonal receptors, such as progesterone (PR) and estrogen (ER) receptors [11,14], of growth factors as VEGF [15], enzymes as COX-2 [4,16], and of telomerase and metalloproteinase activities [12,17].

Tenascin-C (TN-C) is an extracellular matrix (ECM) hexameric glycoprotein composed of six similar subunits [18]. TN-C is more abundantly present in embryonic tissues, like the fetal meninges. It decreases in the adult normal tissues but can increase during pathologic situations, such as wound healing and progression of tumors [19,20]. It is involved in physiological processes such as cell adhesion, proliferation and migration, embryogenesis and organogenesis of many organs, wound healing, and also in pathological situations, as in many neoplasms [18,21]. ECM proteins can act as a substrate for the motility and attachment of neoplastic cells [22]. In humans, the expression of TN-C has been associated with the malignancy of brain glial tumors, such as high-grade astrocytomas [23,24,25]. In this way, previous studies confirm the positive effect of treatment with radiolabeled monoclonal anti-TN-C antibodies [26,27]. A recent investigation also demonstrated a positive relation between human malignant meningiomas and expression of TN-C [28]. Although many studies have analyzed the distribution of different proteins, growth factors, and enzymes in canine meningiomas, there is no research detailing the immunohistochemical pattern of TN-C in this important neoplasm in dogs. The current study aimed to investigate the immunohistochemical distribution of TN-C in different grades of canine meningiomas.

## 2. Materials and Methods

Twenty-one cases of canine meningioma diagnosed by Unitat de Patologia Murina i Comparada (UPMiC) of Universitat Autònoma de Barcelona, Spain, between 2011 and 2020 were analyzed and included in this study. Samples obtained in necropsies and surgical biopsies were included. All cases were evaluated and classified by two neuropathologists (MP and FJFS; MP is board-certified by European College of Veterinary Pathology [ECVP]), according to the WHO’s domestic animal classification [1,10]. All samples were fixed in 10% buffered formalin, processed routinely, and stained with hematoxylin and eosin (HE). In addition, clinical data of each dog, such as sex, breed, age, and anatomical location of the neoplasm, were obtained.

Additional slides were submitted to immunohistochemistry (IHC) using a biotin–peroxidase system and diaminobenzidine as the chromogen. Antigen retrieval was performed with citrate buffer pH 6.0 for 50 min. The slides were incubated in a solution of H_2_O_2_ (3%) in distilled water for 30 min to block the endogenous peroxidase activity. The reagents were applied manually, with an overnight incubation at 4 °C for the monoclonal primary antibodies and a 40 min incubation for the secondary antibodies (Dako, Glostrup, Denmark). The diaminobenzidine chromogen (Dako, Glostrup, Denmark) was applied for 10 min. The IHC antibody panel is described in Table 1. The IHC sections were counterstained using Harris hematoxylin. A sample of the canine normal mammary gland and small intestine was used as the positive control to TN-C and Ki-67, respectively, whereas the skin was used as the control to Pan CK and vimentin. A sample of a canine pulmonary adenocarcinoma was used as a positive control of EMA. For the negative control, an isotype-specific immunoglobulin was used as a substitute for the primary antibody. All IHC protocols were performed on the same day in all cases.

The pattern of the immunohistochemical reaction was graded using a semiquantitative score for the intensity of immunostaining and location of deposits. All samples were evaluated under 20× power magnification (Microscope Nikon, model Eclipse E200 MV R); the deposition of TN-C was graded as follows: score -, no staining; score +, mild (pale immune positivity affecting less than 5% of neoplastic cells area) staining; score ++, moderate (evident immune affecting 5.1–30% of neoplastic cells area) staining; and score +++, severe (strong immune positivity affecting more than 30% of neoplastic cells area) staining. For the other antibodies (EMA, Ki-67, Pan CK, and vimentin), the number of immunolabeled cells was scored based on another study [2]. The images were captured using the NDP.View 2.7.43 software (Hamamatsu Protonics K.K., Shizuoka, Japan)

## 3. Results

Fourteen meningiomas were diagnosed in males and seven in female dogs (Table 2). Mixed breed dogs were the most affected (5/12), followed by the other twelve breeds showing similar distribution. Most cases affected the brain (18/21), and pulmonary metastasis was confirmed only in one case. Adult dogs were predominantly injured, with a median age of 9.75 years.

Seven different types and subtypes of meningioma were evaluated in this study: 12 grade I (3 meningothelial, 2 transitional, 3 psammomatous, 1 fibrous, and 3 microcystic), 6 grade II (atypical), and 3 grade III (papillary) (Table 2).

**Figure 1:**
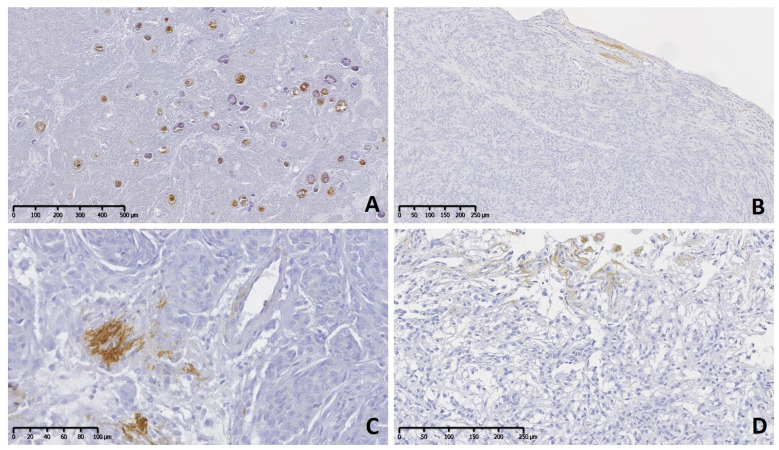
Meningioma grade I, brain, dog. Immunolabeling to tenascin-C (TN-C). (**A**) A psammomatous meningioma shows positive immune reaction in many rounded and concentric whorls of meningothelial cells. (**B**) A transitional subtype with occasional area containing positive extracellular matrix. (**C**) Multifocal stromal areas are mildly positive in meningothelial meningioma. (**D**) A microcystic subtype shows on top a mild immunoreactivity in stroma cells.

**Figure 2:**
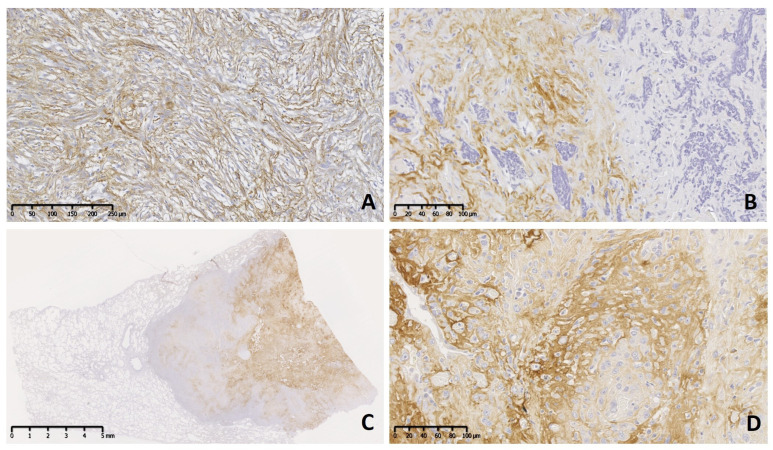
Meningioma grade II (brain) and III (lung [C], brain [D]) dog. Immunolabeling to tenascin-C (TN-C). Two cases of atypical subtype (grade II) present moderate diffuse (**A**) and strong focal (**B**) immunoreactivity in the neoplastic stroma. (**C**) Subgross view of a metastatic meningioma (grade III) affecting the lung with extensive and strong reaction. (**D**) Details of the primary neoplasm show strong immunolabeling in the neoplastic stroma.

The immunohistochemical pattern of TN-C was irregular with mild reaction (+) in grade I (Figure 1A–D), predominantly moderate (++) in grade II, and moderate to severe (++–+++) in grade III neoplasms (Figure 2A–D). Usually, immune positivity was observed mainly in the stroma (extracellular space) and perivascular spaces in all grades and subtypes. In addition, the concentric whorls of neoplastic cells were labeled clearly positive in a few psammomatous (Figure 1A) and transitional meningiomas (Figure 1B). In grade I cases, the neoplastic cell areas were rarely immunolabeled to TN-C, with the exception of one fibrous subtype that presented score II of stromal and cellular reactivity. The reaction to TN-C was more significant in grade II and III tumors, mainly in the stroma, perivascular spaces, and necrotic areas (Figure 2A–D). Due to the abnormally high expression of TN-C observed in one case initially morphologically classified as a fibrous meningioma, this case was considered a grade II atypical meningioma (Figure 2A).

Histopathological diagnosis of meningioma was reinforced with the immune positivity to vimentin (moderate to strong) and EMA (mild to moderate) in neoplastic cells in most cases, independently of its grade or subtype. Immune positivity for Ki-67 was observed in 1 to 5% of neoplastic cells (+) in most grade I cases, between 15 and 40% neoplastic cells (++) in three grade II cases, and 50–70% neoplastic cells (+++) in two grade III cases. Pan CK reactivity was predominantly negative (14/21) or mild (6/21) in the neoplastic cells. Only one grade II case showed approximately 85% positivity to Pan CK.

## 4. Discussion

Meningioma is frequently diagnosed in the brain of adult dogs with a median age of 9–10 years [1,2,6]. Similar data were observed in the current study. Some breeds, such as Boxer, Golden Retriever, and German Shepherd, seem to be more predisposed to meningioma [2,6,8]. In the present study, dogs of mixed breeds were more affected. Apparently, there is no sex predisposition to meningioma in animals, unlike what occurs in humans, where women are more affected by this neoplasm [1,2,6]. Although surgical removal is the primary therapeutic option in canine meningiomas, sometimes associated with chemotherapy and radiotherapy, corticosteroids and antiepileptic drugs can also be used [3]. These therapeutic options can improve the clinical signs of affected dogs. Differential diagnoses of meningioma in dogs include other neoplasms with extra-axial locations, such as histiocytic sarcoma, metastatic carcinomatosis, and granular cell tumor [1].

Although some studies have investigated the expression of TN-C in human brain neoplasms, there are no similar studies in canine neoplasms affecting the central nervous system (CNS). For the first time, a study analyzed the immunohistochemical expression of TN-C in canine meningioma and compared it with those previously evaluated in humans [19,28,29,30]. The current study demonstrated that TN-C reactivity is strongly expressed mainly in more aggressive meningiomas (grade II and III cases), as similarly visualized in anaplastic forms of human meningiomas [19,29]. However, it would be necessary to evaluate a larger number of grade II and III meningiomas to associate the positive immune expression of TN-C with its biological behavior. In humans, TN-C is considered to be a stromal marker in perivascular spaces and mechanically resistant areas of meningiomas. In the current study, the reaction was mild and restricted to stroma and perivascular space in grade I tumors, independently of its histopathological subclassification. Very similar data were detected in an immunohistochemical study that analyzed 69 human meningiomas [19]. Another study analyzing human meningiomas concluded that TN-C expression is correlated with anaplasia, tumor-associated edema, and VEGF expression but not with tumor border shape in meningiomas [29]. In the other hand, immune-positive reactivity to TN-C was also observed in the stroma and less commonly in neoplastic cells of other human brain neoplasms, such as gliomas [19,25,28]. In a study of canine mammary neoplasia, immunoreactivity to TN-C was also observed in malignant neoplastic epithelial cells, suggesting that these cells can synthesize ECM components [31]. Another previous study analyzed TN-C expression in the cartilaginous tissue of canine mammary mixed tumors [32]. In addition, tenascin has been expressed intensively in the capillary basement membranes and stroma of other human anaplastic neoplasms, such as melanomas, fibrosarcoma, and mammary carcinoma [18,23]. Based on these findings observed on human meningiomas, in the present study, one case morphologically classified as grade I meningioma, fibrous subtype, was reclassified as atypical meningioma (grade II) due to its moderate immunoreactivity to TN-C. The current study’s immunohistochemical findings indicate no direct relation of TN-C reactivity compared to other antibodies (vimentin, EMA, Pan CK, Ki-67).

More information is still needed about the exact mechanism of the role of TN-C in the progression of neoplasia. However, the pattern of immunohistochemical reactivity suggests that TN-C can act as a poor prognostic factor to aggressive human meningiomas. Although the results of the current study showed stronger immunoreaction in grade II and III neoplasms, this analysis would be reinforced with more tested cases. The findings of the present study indicate that TN-C is a good stromal marker in canine meningioma, but its real biological role in dogs needs to be further investigated. Furthermore, more studies in dogs could evaluate if this ECM glycoprotein would be considered a potential target for meningioma therapy, as previously speculated in humans [29]. It is possible that TN-C can be involved in an anti-adhesion mechanism that can help the neoplastic cells proliferate in the adjacent CNS [30]. Studies indicate that TN-C seems to be modulated similarly in human and canine neoplasms [20]. Future studies are necessary to determine other possible pathogenic mechanisms related to the action of TN-C in the tumoral biology of canine meningioma.

## Figures and Tables

**Table 1:** Panel of the antibodies used in the current study.

Antibody	Clone	Manufacter	Dilution
Tenascin-C	4F10TT	IBL, Fujioka, Japan	1:100
EMA	E29	Dako, Glostrup, Denmark	1:40
Ki-67	B56	BD Pharmingen, Franklin Lakes, NJ, USA	1:100
Pan CK	MNF116	Dako, Glostrup, Denmark	1:100
Vimentin	V9	Dako, Glostrup, Denmark	1:200

**Table 2:** Clinical data, anatomical sites, and immunohistochemical pattern in 21 cases of canine meningioma.

Case	Classification	Age (Years)	Breed	Sex	Location	TN-C	EMA	Ki-67	PanCK	Vimentin
1	Meningothelial—I	8	Maltese	M	B	+	+++	+	+	+++
2	Meningothelial—I	11	West Highland White Terrier	F	B	+	+	++	-	+++
3	Meningothelial—I	10	Mixed breed	M	B	+	+++	++	+	+++
4	Transitional—I	3	Maltese	M	SC	+	+++	+	-	+++
5	Transitional—I	11	Belgian Shepherd	F	B	+	+++	+	-	+++
6	Psammomatous–I	11	Cocker Spaniel	F	B	+	+++	+	-	+++
7	Psammomatous–I	7	German Shepherd	F	B	-	+++	+	-	+++
8	Psammomatous–I	7	Mixed breed	M	B	+	+++	+	-	+++
9	Fibrous—I	14	Poodle	F	B	+	+++	+	-	+++
10	Microcystic—I	9	Mixed breed	F	SC	+	+	+	-	+
11	Microcystic—I	6	Rhodesian Ridgeback	M	SC	+	+++	++	+	++
12	Microcystic—I	10	Golden Retriever	M	B	+	+++	+	+	++
13	Atypical—II	8	German Shepherd	M	SC	++	+++	+	-	+++
14	Atypical—II	12	Miniature Schnauzer	M	B	++	+++	++	-	+++
15	Atypical—II	n.i.	Mixed breed	F	B	+	+++	+	+	+++
16	Atypical—II	12	Golden Retriever	M	B	++	+++	+	-	+++
17	Atypical—II	12	Boxer	M	B	-	+++	+	-	+++
18	Atypical—II	11	Golden Retriever	M	B	++	+	+++	-	++
19	Papillary—III	8	Mixed breed	M	B	++	+++	+	-	+++
20	Papillary—III	12	Labrador Retriever	M	B	++	+++	+++	+	+++
21	Papillary—III	13	Spanish Breton	M	B, L	+++	+++	++	-	+

M—male, F—female, TN-C—tenascin C, EMA—epithelial membrane antigen, Pan CK—pan cytokeratin, SC—spinal cord, B—brain, L—lung.

## Data Availability

The data that support the findings of this study are available from the corresponding author upon reasonable request.

## References

[1] Higgins R.J., Bollen A.W., Dickinson P.J., Sisó-Llonch S., Meuten D.J. (2017). Tumors of the nervous system. Tumors in Domestic Animals.

[2] Montoliu P., Añor S., Vidal E., Pumarola M. (2006). Histological and immunohistochemical study of 30 cases of canine meningioma. J. Comp. Pathol..

[3] Motta L., Mandara M.T., Skerritt G.C. (2012). Canine and feline intracranial meningiomas: An updated review. Vet. J..

[4] Samarani F., de la Fuente C., Parodi A., Mandara M.T., Pumarola M., Añor S. (2018). Immunohistochemical expression of cyclooxygenase-2 (COX-2) is not associated with tumor grade in feline meningiomas. Vet. J..

[5] O’Rahilly R., Müller F. (1986). The meninges in human development. J. Neuropathol. Experim. Neurol..

[6] Sturges B.K., Dickinson P.J., Bollen A.W., Koblik P.D., Kass P.H., Kortz G.D., Vernau K.M., Knipe M.F., Lecouteur R.A., Higgins R.J. (2008). Magnetic resonance imaging and histological classification of intracranial meningiomas in 112 dogs. J. Vet. Intern. Med..

[7] Patnaik A.K., Lieberman P.H., Erlandson R.A., Shaker E., Hurvitz A.I. (1986). Paranasal meningioma in the dog: A clinicopathologic study of ten cases. Vet. Pathol..

[8] Snyder J.M., Shofer F.S., Van Winkle T.J., Massicotte C. (2006). Canine intracranial primary neoplasia: 173 cases (1986–2003). J. Vet. Intern. Med..

[9] Greco J.J., Aiken S.A., Berg J.M., Monette S., Bergman P.J. (2006). Evaluation of intracranial meningioma resection with a surgical aspirator in dogs: 17 cases (1996–2004). J. Am. Anim. Hosp. Assoc..

[10] Vandevelde M., Higgins R.J., Oevermann A. (2012). Veterinary Neuropathology, Essentials of Theory and Practice.

[11] Mandara M.T., Ricci G., Rinaldi L., Sarli G., Vitellozzi G. (2002). Immunohistochemical identification and image analysis quantification of oestrogen and progesterone receptors in canine and feline meningioma. J. Comp. Pathol..

[12] Mandara M.T., Pavone S., Mandrioli L., Bettini G., Falzone C., Baroni M. (2009). Matrix metalloproteinase-2 and matrix metalloproteinase-9 expression in canine and feline meningioma. Vet. Pathol..

[13] Matiasek L.A., Platt S.R., Adams V., Scase T.J., Keys D., Miller J., Adamo F., Long S., Matiasek K. (2009). Ki-67 and vascular endothelial growth factor expression in intracranial meningiomas in dogs. J. Vet. Intern. Med..

[14] Théon A.P., LeCouteur R.A., Carr E.A., Griffey S.M. (2000). Influence of tumor cell proliferation and sex-hormone receptors on effectiveness of radiation therapy for dogs with incompletely resected meningiomas. J. Am. Vet. Med. Assoc..

[15] Platt S.R., Scase T.J., Adams V., Wieczorek L., Miller J., Adamo F., Long S. (2006). Vascular endothelial growth factor expression in canine intracranial meningioma and association with patient survival. J. Vet. Intern. Med..

[16] Rossmeisl Jr J.H., Robertson J.L., Zimmerman K.L., Higgins M.A., Geiger D.A. (2009). Cyclooxygenase-2 (COX-2) expression in canine intracranial meningiomas. Vet. Comp. Oncol..

[17] Mandrioli L., Panarese S., Cesari A., Mandara M.T., Marcato P.S., Bettini G. (2007). Immunohistochemical expression of h-telomerase reverse transcriptase in canine and feline meningiomas. J. Vet. Sci..

[18] Koukoulis G.K., Gould V.E., Bhattacharyya A., Gould J.E., Howeedy A.A., Virtanen I. (1991). Tenascin in normal, reactive, hyperplastic and neoplastic tissues: Biologic and pathologic implications. Hum. Pathol..

[19] Castellani P., Dorcaratto A., Siri A., Zardi L., Viale G.L. (1995). Tenascin distribution in human brain tumors. Acta Neurochir..

[20] Mukaratirwa S., Nederbragt H. (2002). Tenascin and proteoglycans: The role of tenascin and proteoglycans in canine tumours. Res. Vet. Sci..

[21] Yoshimura H., Michishita M., Ohkusu-Tsukada K., Matsuda Y., Ishiwata T., Naito Z., Takahashi K. (2015). Cellular sources of tenascin-C in canine mammary carcinomas. Vet. Pathol..

[22] Goldbrunner R.H., Bernstein J.J., Tonn J.C. (1999). Cell-extracellular matrix interaction in glioma invasion. Acta Neurochir..

[23] Bourdon M.A., Wikstrand C.J., Furthmayr H., Matthews T.J., Bigner D.D. (1983). Human glioma-mesenchymal extracellular matrix antigen defined by monoclonal antibody. Cancer Res..

[24] Hasegawa K., Yoshida T., Matsumoto K., Katsuta K., Waga S., Sakakura T. (1997). Differential expression of tenascin-C and tenascin-X in human astrocytomas. Acta Neuropathol..

[25] Higushi M., Ohnishi T., Arita N., Hiraga S., Hayakawa T. (1993). Expression of tenascin in human gliomas: Its relation to histological malignancy, tumor dedifferentiation and angiogenesis. Acta Neuropathol..

[26] Reardon D.A., Akabani G., Coleman R.E., Friedman A.H., Friedman H.S., Herndon J.E., Cokgor I., McLendon R.E., Pegram C.N., Provenzale J.M. (2002). Phase II trial of murine ^131^I-labeled antitenascin monoclonal antibody 81C6 administered into surgically created resection cavities of patients with newly diagnosed malignant gliomas. J. Clin. Oncol..

[27] Riva P., Franceschi G., Frattarelli M., Lazzari S., Riva N., Giuliani G., Casi M., Guiducci G., Giorgetti G., Gentile R. (1999). Loco-regional radioimmunotherapy of high-grade malignant gliomas using specific monoclonal antibodies labeled with 90Y: A Phase I study. Clin. Cancer Res..

[28] Leins A., Riva P., Lindstedt R., Davidoff M.S., Mehraein P., Weis S. (2003). Expression of Tenascin-C in Various Human Brain Tumors and its Relevance for Survival in Patients with Astrocytoma. Cancer.

[29] Kiliç T., Bayri Y., Özduman K., Acar M., Diren S., Kurtkaya O., Ekinci G., Bugra K., Sav A., Ozek M.M. (2002). Tenascin in mengioma: Expression is correlated with anaplasia, vascular endothelial growth factor expression, and peritumoral edema but not with tumor border shape. Neurosurgery.

[30] Montagnani S., Castaldo C., Di Meglio F., Sciorio S., Giordano-Lanza G. (2000). Extra cellular matrix features in human meninges. Ital. J. Anat. Embryol..

[31] Faustino A.M., van Garderen E., Schalken J.A., Nederbragt H. (2002). Tenascin expression in normal, hyperplastic, dysplastic and neoplastic canine mammary tissues. J. Comp. Pathol..

[32] Arai K., Naoi M., Uehara K. (1994). Immunohistochemical examination of neural cell adhesion molecule (NCAM), tenascin and fibronectin on the development of cartilaginous tissue in mammary mixed tumors. J. Vet. Med. Sci..

